# Limb volume measurements: comparison of accuracy and decisive parameters of the most used present methods

**DOI:** 10.1186/s40064-015-1468-7

**Published:** 2015-11-19

**Authors:** Adam Chromy, Ludek Zalud, Petr Dobsak, Igor Suskevic, Veronika Mrkvicova

**Affiliations:** International Clinical Research Center, St. Anne’s University Hospital Brno, Brno, Czech Republic; Central European Institute of Technology, Brno University of Technology, Technicka 3082/10, 616 00 Brno, Czech Republic; Faculty of Electrical Engineering and Communication, Brno University of Technology, Brno, Czech Republic; Department of Preventive Medicine, Faculty of Medicine, Masaryk University of Brno, Brno, Czech Republic

**Keywords:** Volumetric measurements, Volumetric methods, Body volume

## Abstract

Limb volume measurements are used for evaluating growth of muscle mass and effectivity of strength training. Beside sport sciences, it is used e.g. for detection of oedemas, lymphedemas or carcinomas or for examinations of muscle atrophy. There are several commonly used methods, but there is a lack of clear comparison, which shows their advantages and limits. The accuracy of each method is uncertainly estimated only. The aim of this paper is to determine and experimentally verify their accuracy and compare them among each other. Water Displacement Method (WD), three methods based on circumferential measures—Frustum Sign Model (FSM), Disc Model (DM), Partial Frustum Model (PFM) and two 3D scan based methods Computed Tomography (CT) and Magnetic Resonance Imaging (MRI) were compared. Precise reference cylinders and limbs of two human subjects were measured 10 times by each method. Personal dependency of methods was also tested by measuring 10 times the same object by 3 different people. Accuracies: WD 0.3 %, FSM 2–8 % according person, DM, PFM 1–8 %, MRI 2 % (hand) or 8 % (finger), CT 0.5 % (hand) or 2 % (finger);times: FSM 1 min, CT 7 min, WD, DM, PFM 15 min, MRI 19 min; and more. WD was found as the best method for most of uses with best accuracy. The CT disposes with almost the same accuracy and allows measurements of specific regions (e.g. particular muscles), as same as MRI, which accuracy is worse though, but it is not harmful. Frustum Sign Model is usable for very fast estimation of limb volume, but with lower accuracy, Disc Model and Partial Frustum Model is useful in cases when Water Displacement cannot be used.

## Background

Since very beginning of sport, visual observation of limb volume has been the most common method evaluating growth of muscle mass (De Santo et al. 2011), what consequently evaluates effectivity and utility of applied strength training schedule (Silva-Couto et al. 2014).

But if you are *observing only*, the changes in muscle mass are visible only when increments or decrements are significantly large, e.g. after longer exercising period. To be able to detect the efficiency of training in its *very beginning* or to be able to *objectively compare* two training methods, we have to detect *tiny differences* of muscle mass. In this case, the observing is insufficient—it is necessary to *measure* volumetric changes of limb (Akagi et al. 2009; Kaulesar Sukul et al. 1993; Knarr et al. 2013).

Beside sport sciences, the measurement of limb volume with sufficient precision is also valuable for many other purposes—e.g. for early detection of peripheral oedemas (Brijker et al. 2000; Haase et al. 2009; Haponiuk et al. 2013), lymphedemas, carcinomas (Ridner et al. 2007) or fibrosis (Ribeiro et al. 2010), its monitoring and control of its evolution; measurement of rehabilitation progress (Khanavi et al. 2014; Konecny 2013), measurements of muscle atrophy (Ramsay et al. 2011; Silva-Couto M de et al. 2014) or supervision of recovery process after invasive surgeries (Konecny 2013; Sproule et al. 2011; Wachal et al. 2014).

According to (Armer and Ridner 2006; Brijker et al. 2000; Kaulesar Sukul et al. 1993; Lavelle and Stanton 2014; Ridner et al. 2007), the presently most used methods intended for measurements of limb volumes are: circumferential methods called *Frustum Sign Model*, *Disc Model* and their conjunction *Partial Frustum Model*; method called *Water Displacement Volumetry* and methods based on 3D model provided by *Magnetic Resonance Imaging* (MRI) or *Computed Tomography* (CT). There are several papers about their practical use, but there is not enough information about their accuracy (only uncertain estimations) and their specific advantages and limits.

The aim of this paper is to determine and experimentally verify their accuracy and compare them among each other also in other parameters, which are decisive to their usability. The result of this work should be an objective overview of available methods and should serve as guide when choosing the proper method for particular application.

## Methods

The first part of this section describes reference objects used for the following experiments. In the second part, measuring procedure of each tested method is described. Final part describes both comparative experiments: accuracy and repeatability verification experiment and personal dependency test.

### Reference objects

**Fig. 1:**
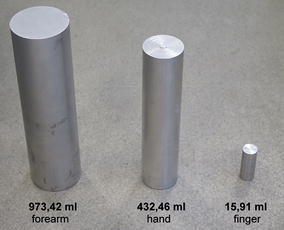
Precise reference cylinders for verification of accuracy of Water Displacement Method and circumferential methods

For verification of accuracy of Water Displacement Method and the circumferential measurements, we use precise aluminium cylinders in three sizes (Fig. 1) with volumes similar to finger, hand and forearm. Dimensions of each reference cylinder has been measured with slide calliper and according to (Jamerson 2009) their volumes were computed as 15.91 ± 0.06, 432.46 ± 0.53 and 973.42 ± 0.89 ml.

**Fig. 2:**
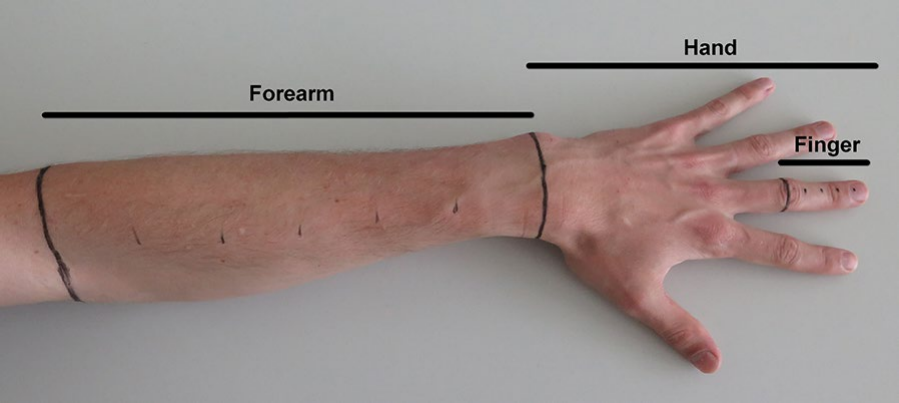
Patient’s upper limb with marked region of interest—forearm, hand and finger regions

For verification of accuracy of Magnetic Resonance Imaging and Computed Tomography, as same as for comparative experiments among different methods, two real human limbs were used. At patient’s limb, borders of three regions of interest were marked with permanent marker (Fig. 2) as follows:

#### Finger

Region situated at middle finger of left hand, in distal direction from axial cutting plane located at the centre of proximal interphalangeal joint (*articulatio interphalangealis proximalis digii tertii*).

#### Hand

Region of left hand, in distal direction from axial cutting plane going through both ulnar styloid process (*processus styloideus ulnae*) and radial styloid process (*processus styloideus radii*).

#### Forearm

Region of forearm between axial cutting plane going through both ulnar styloid process (*processus styloideus ulnae*) and radial styloid process (*processus styloideus radii*) and axial cutting plane going through olecranon and cubital fossa (*fossa cubitalis*).

Each region of interest has been defined as above because of the fact that MRI and CT modalities do not recognize the border defined with permanent marker, so we have to be able to exactly determine boundaries of region just from the image of bones inside the limb, which are clearly visible at CT and MRI images.

Note: When words Finger, Hand and Forearm are written in following text with first character in capital, it is used in mean of the region of interest defined above, not in its anatomical sense.

### Water Displacement Method

The most commonly used volumetric method is based on quantum of water overflowing from fully filled container when measured limb is inserted (Armer and Ridner 2006; Megens et al. 2001; Szopinski et al. 2014).

**Fig. 3:**
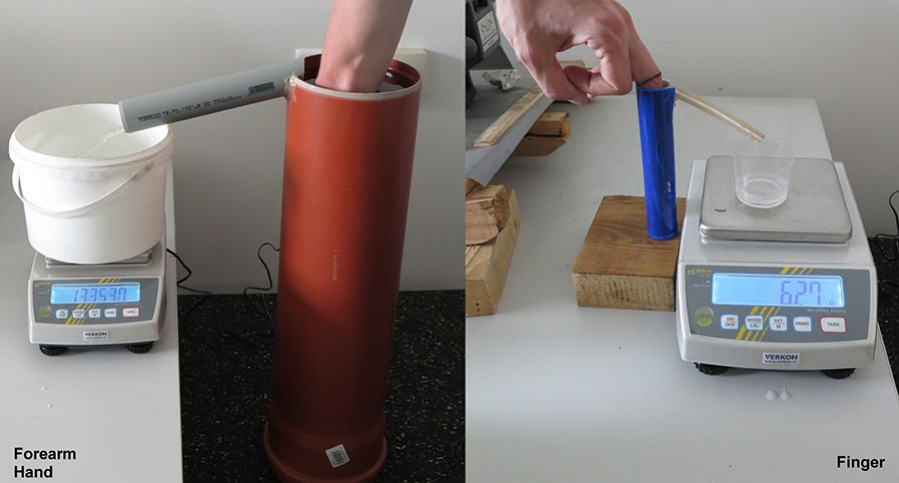
The measuring apparatus for Water Displacement Method. Two water tubes with different sizes for various sizes of measured object

The experimental apparatus is shown on Fig. 3. It consists of concave tube, closed at the bottom side and equipped with spillway on the top of the tube (Lavelle and Stanton 2014). The spillway fall into the container placed at the precise digital weight scale KERN PCB 2500-2 (d = 0.01 g). There are two different tubes used in this experiment: the smaller one has a diameter 27 mm and the height 150 mm and is intended for measurements of small objects like a finger. The bigger one has a diameter 156 mm and the height 595 mm and is intended for measurements of objects in size of hand or forearm.

At the start of measurement, the container is emptied and tube is filled with water since water starts to float through the spillway. We wait so long as the spilled water fully drops away (up to 10 min at bigger tube and 20 s at smaller tube) and reset the scale value (TARE). Then, the measured object is inserted into the tube up to the marked edge of region of interest (in case of human limb) or fully drowns in case of reference cylinder. We wait so long as the spilled water fully drops away (in case of reference cylinder) or when the dropping period is longer than 1 s (in case of human limb, because the shivering of limb vibrates with water level and dropping of water does not ceases totally). The weight of spilled water in grams is the value of volume in millilitres, since the density of water is 0.999 g/ml at 20 °C (Cmelik et al. 2011).

The method is frequently used because of its simplicity and very high accuracy. The main disadvantages are, that it requires good flexibility of measured limb, good motoric functions of patient (shivering of limb significantly influences result) and it is very time consuming (Damstra 2009; Deltombe et al. 2007; Kaulesar Sukul et al. 1993; Lavelle and Stanton 2014; Ridner et al. 2007). Region of interest is limited to level of immersion only, what is not suitable for specific measurements (e.g. size of particular muscle).

### Frustum Sign Model

**Fig. 4:**
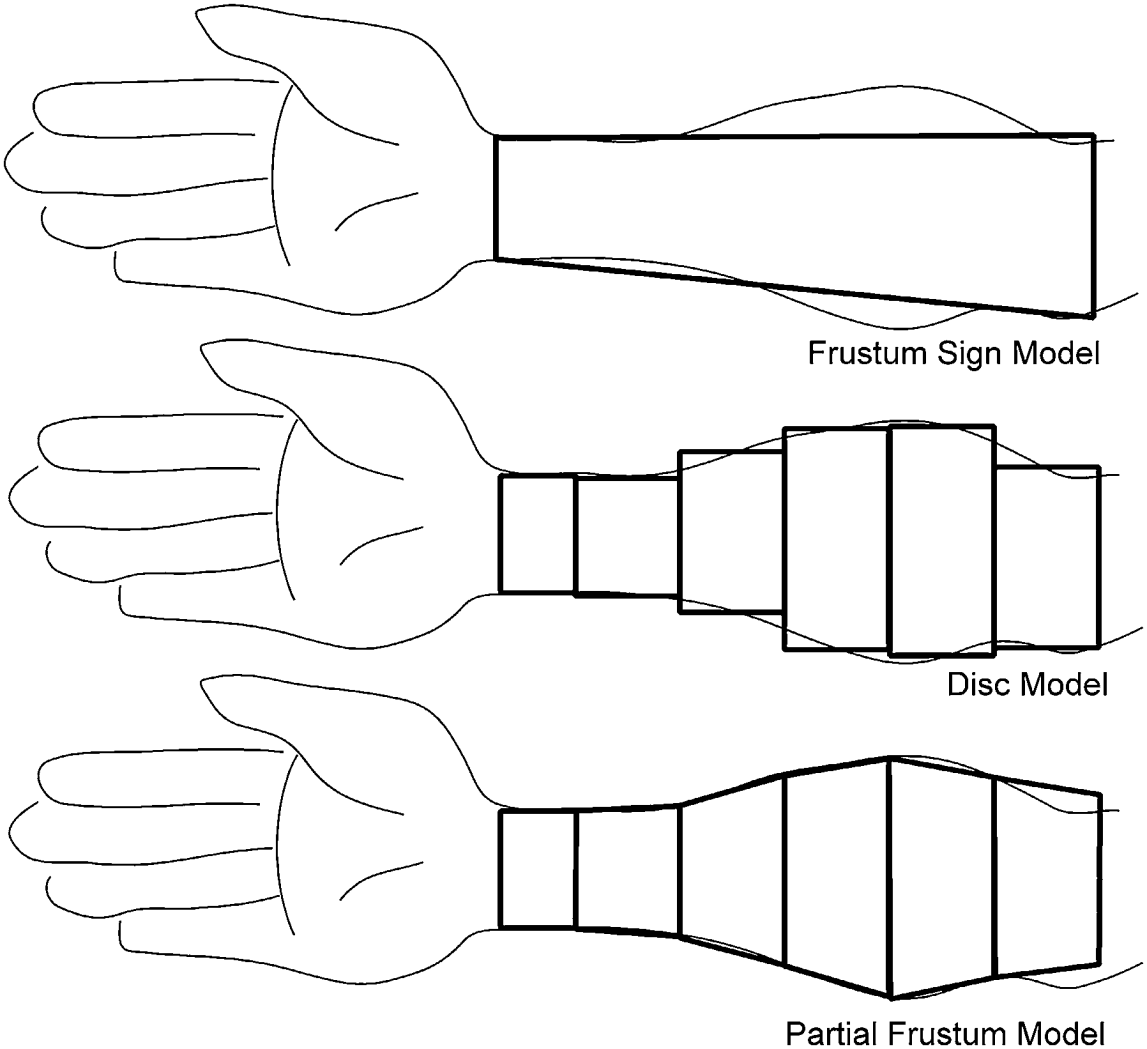
Schematic difference between the real volume of Forearm region and particular circumferential approximations

The experimental apparatus consists of the non-elastic string with diameter d_s_ = 3.45 mm and the ruler with d = 1 mm. There are just 2 circumferential measurements taken at opposite sides of measured region and the volume of limb is approximated by truncated cone between them (Fig. 4) (Deltombe et al. 2007).

Instead of standard equations from (Armer and Ridner 2006; Deltombe et al. 2007; Ridner et al. 2007), we used its modification, since the diameter of measuring string indispensably influences the result:

$$ V_{F} = \frac{\pi }{3} \cdot h \cdot \left[ {\left( {\frac{{C_{1} }}{2\pi } - \frac{{d_{s} }}{2}} \right)^{2} + \left( {\frac{{C_{1} }}{2\pi } - \frac{{d_{s} }}{2}} \right) \cdot \left( {\frac{{C_{2} }}{2\pi } - \frac{{d_{s} }}{2}} \right) + \left( {\frac{{C_{2} }}{2\pi } - \frac{{d_{s} }}{2}} \right)^{2} } \right] $$

where *h* is distance between two circumferential measurements, *C*_*1*_ and *C*_*2*_ are measured values of circumference and *d*_*s*_ is diameter of measuring string.

**Fig. 5:**
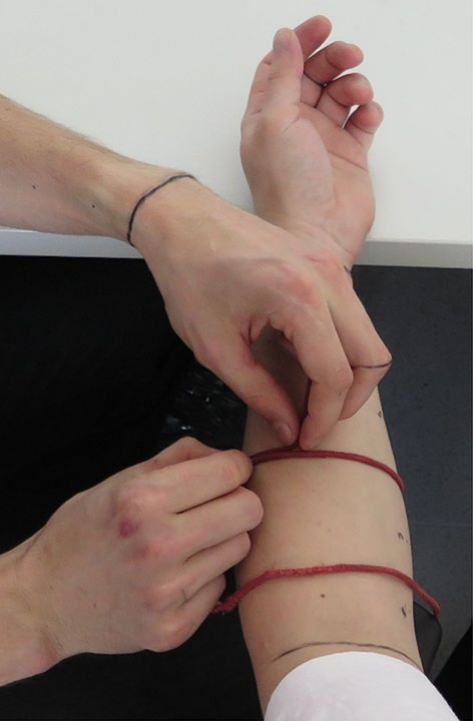
Measuring of limb circumference used at Frustum Sign Model, Disc Model and Partial Frustum Model method

The patients were placed in a sitting position with forearms pronated. The string was placed around the arm; always in direct contact with the skin but without excessive pressure (Fig. 5) and the circumference was marked on the string. The length of marked part of string was measured by ruler. Measurements of arm circumference were captured at the level of both defining cutting planes of Forearm region, in case of Finger, the first circumference was taken at defining cutting plane and the second one was taken 10 mm proximally from the tip of middle finger. The measurements of Hand region were not performed, since the method is not intended for this purpose.

This method is primarily used in cases, when the measured limb is not flexible enough to be placed into the water, in case of water-incompatible disease or in case, when patient limb is shivering too much (Ridner et al. 2007). Provides very quick and easy measurements, but its accuracy is low (Deltombe et al. 2007) and the results significantly depend on personal experiences of staff (Armer and Ridner 2006; Karakas and Bozkir 2012). The possibility of region of interest selection is also very limited.

### Disc Model

The experimental apparatus and the method procedure is the same as in case of Frustum Sign Model, the only difference is that circumferential measurements are taken each 40 mm (Forearm region) or 10 mm (Finger region) from proximal border cutting plane of region and the total volume is computed as sum of equidistant discs (Fig. 5) (Deltombe et al. 2007).

We use the modification of equation by (Kaulesar Sukul et al. 1993), because the diameter of measuring string influences the result and cannot be neglected:

$$ V_{D} = \mathop \sum \limits_{n} \pi \cdot h \cdot \left( {\frac{C}{2\pi } - \frac{{d_{s} }}{2}} \right)^{2} $$

where *h* is distance between two circumferential measurements, *C* is measured value of circumference and *d*_*s*_ is diameter of measuring string.

This method has the same advantages and usage purposes as Frustum Sign Model, but is a bit more accurate (Kaulesar Sukul et al. 1993; Sander et al. 2002), however more time consuming (Haase et al. 2009).

### Partial Frustum Model

This method combines two methods mentioned above. It takes the same measurements as Disc Model, but approximates volume by 40 mm or 10 mm high truncated cones instead of equidistant discs (Fig. 5) (Sander et al. 2002). The equation used at Frustum Sign Model was used.

This method has the same advantages and usage purposes as two previous methods, but it is slightly more time consuming compare to Disc Model if counted manually, but in case of computer processing, the time is the same and the accuracy is better since the approximation is more relevant to real volume (Lavelle and Stanton 2014).

### Magnetic Resonance Imaging (MRI)

MRI is an imaging modality providing 3D models of human body, including their inner structures (Udupa and Herman 1999; Webb 2003). Although common volumetric application of MRI are mostly focused on measurements of inner organs (Chlosta et al. 2011; Gaszynski and Szewczyk 2014), MRI is used for limb volume measurements too (Akagi et al. 2009; Hackney et al. 2012; Knarr et al. 2013; Ramsay et al. 2011; Silva-Couto M de et al. 2014).

Its significant advantage is the possibility of selection of arbitrary regions of interest, what allows measurements of particular muscles, ligaments or bones instead of entire limb only as in case of previous methods (Udupa and Herman 1999). Nevertheless, the method is not frequently used because of its important disadvantages –high acquisition and operational costs (“Magnetic Resonance Imaging,” 2010), time consuming measurement procedure (Seidl and Vaněčková 2007) and limitation of patients with pacemakers or piercing (Novelline and Squire 2004).

The experimental apparatus consists of GE Discovery MR750 3T magnetic resonance imager providing captured data in DICOM format and open source software 3D Slicer (“3D Slicer,” 2015) capable of processing this data.

The patient’s body was situated in pronated position, with left upper limb raised upwards, shoulder joint in flexion, with forearms pronated. Region of hand was placed inside of measuring area of MRI device. Measurement using Ax T1 FSPGR 3D protocol was performed with Slice Thickness of 1.4 mm. The Hand region only was scanned due to the financial reason.

**Fig. 6:**
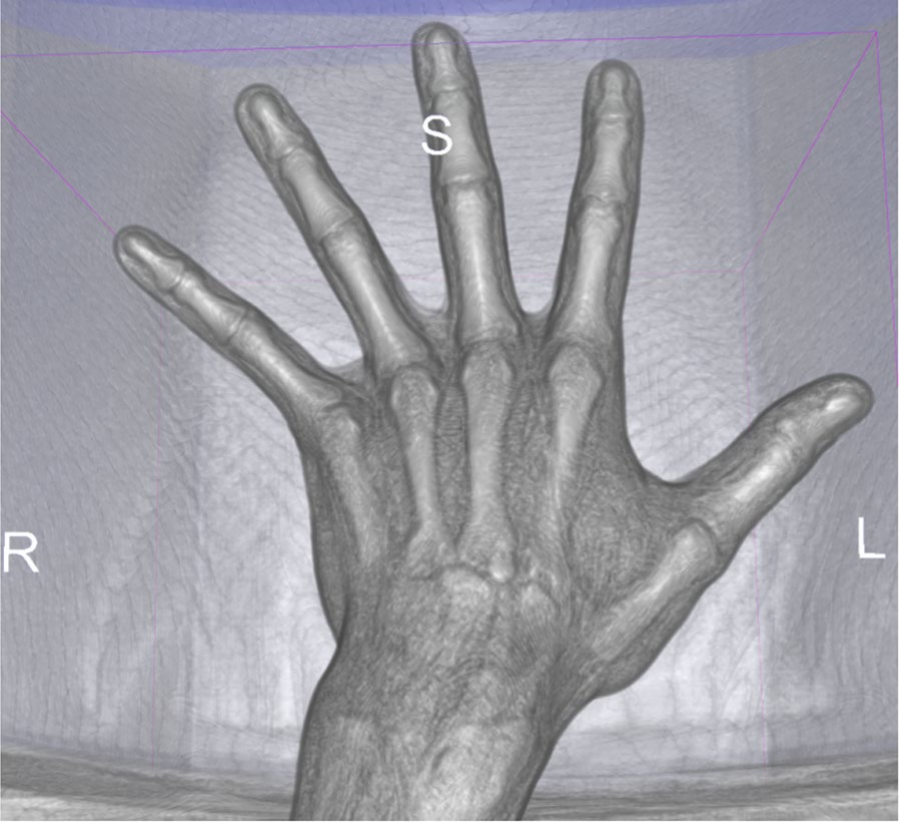
Visualization of captured 3D data from CT in 3D Slicer software. Using opaque tissues volume rendering to be able to see the bones because of precise setting of the region of interest

Scanned 3D data (Fig. 6) were processed in 3D Slicer as follows: measured region was cropped by cutting planes going through the exact points at skeleton as defined above. Using Threshold Effect tool of Editor, we created label map containing volume of measured region. Using Dilate and Erode effect, we clean the unlabelled islands inside of region. Finally, the volume of labelled area has been computed from number of labelled voxels and known size of voxel (Spinczyk, 2014).

### Computed Tomography (CT)

CT uses x-ray instead of magnetic spin in order to build the 3D model, but the volume is computed the same way as MRI—from 3D model provided by imaging modality. Compare to MRI, the CT provides better contrast, lower noise and higher spatial resolution (Herman 2009), what leads to better accuracy (McCollough and Zink 1999). The operational costs are also significantly lower (Udupa and Herman 1999). On the other hand, the ionizing radiation absorbed by patient during average scan can be up to 15 mSv (whole body scanning) (Trends et al. 2008), what is one-third of allowed exposition for workers with ionizing source per year and exceeds Czech generic hygiene limits for common people even 15 times (Statni urad pro jadernou bezpecnost 2002). For this reason, the use of this modality is allowed as rare as possible and repeated scanning is out of the question.

The patient was situated in the same position as at MRI. Measurement with Slice Thickness of 0.6 mm was performed. The Hand region only was scanned due to the ionizing radiation and financial reason.

Data processing was performed the same way as at MRI.

### Accuracy and repeatability experiments

**Fig. 7:**
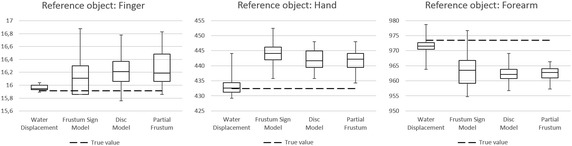
Results of comparison among circumferential methods and Water Displacement method performed on reference object with known volume

Since there is no universal object, which volume can be exactly computed and at the same time it is measurable by all compared methods, the experiments were processed as follows:

*In first step*, the accuracy of Water Displacement and all three circumferential methods has been verified on precise reference cylinders with known volume. Each cylinder was measured using each method 10 times (by the same person) in order to determine its repeatability. Results of this step are shown in Table 1 in rows 1, 4 and 7 and on Fig. 7. These results served for assessment of accuracy of Water Displacement, Frustum Sign Method, Disc Model and Partial Frustum.

**Table 1:** Overview of result values and their standard deviations measured using different methods

	Method	Water Displ.		Frustum Sign		Disc Model		Partial Frustum		MRI		CT	
	Measured object Reference value	Measured value RSD/RACC		Measured value RSD/RACC		Measured value RSD/RACC		Measured value RSD/RACC		Measured value RSD/RACC		Measured value RSD/RACC	
1	Reference finger 15.91 ± 0.06 ml	15.96 ± 0.04 ml		16.14 ± 0.31 ml		16.22 ± 0.27 ml		16.27 ± 0.28 ml		NA		NA	
		0.3 %	0.3 %	1.9 %	1.4 %	1.7 %	1.9 %	1.7 %	2.2 %				
2	Finger, subject 1 13.80 ± 0.34 ml	13.80 ± 0.34 ml		13.66 ± 0.78 ml		13.08 ± 0.59 ml		12.66 ± 0.58 ml		13.22 ± 0.62 ml		13.41 ± 0.28 ml	
		2.5 %		5.7 %	1.0 %	4.5 %	5.2 %	4.6 %	8.2 %	4.7 %	4.2 %^a^	2.1 %	2.8 %^a^
3	Finger, subject 2 13.69 ± 0.21 ml	13.69 ± 0.21 ml		14.62 ± 0.39 ml		13.71 ± 0.37 ml		13.11 ± 0.35 ml		14.81 ± 0.70 ml		13.88 ± 0.35 ml	
		1.6 %		2.7 %	6.8 %	2.7 %	0.2 %	2.7 %	4.2 %	4.7 %	8.2 %^a^	2.5 %	1.4 %^a^
4	Reference hand 432.46 ± 0.53 ml	433.74 ± 4.12 ml		443.82 ± 5.04 ml		441.63 ± 3.86 ml		441.53 ± 3.93 ml		NA		NA	
		0.9 %	0.3 %	1.1 %	2.6 %	0.9 %	2.1 %	0.9 %	2.1 %				
5	Hand, subject 1 397.70 ± 4.84 ml	397.70 ± 4.84 ml		NA		NA		NA		392.27 ± 18.71 ml		399.49 ± 6.54 ml	
		1.2 %								4.8 %	1.4 %^a^	1.6 %	0.4 %^a^
6	Hand, subject 2 439.10 ± 7.66 ml	439.10 ± 7.66 ml		NA		NA		NA		428.79 ± 21.46 ml		439.54 ± 7.34 ml	
		1.7 %								5.0 %	2.3 %^a^	1.7 %	0.1 %^a^
7	Reference forearm 973.42 ± 0.89 ml	971.47 ± 3.50 ml		963.95 ± 6.35 ml		962.49 ± 3.78 ml		962.37 ± 2.83 ml		NA		NA	
		0.4 %	0.2 %	0.7 %	1.0 %	0.4 %	1.1 %	0.3 %	1.1 %				
8	Forearm, subject 1 1184.90 ± 9.91 ml	1184.90 ± 9.91 ml		1087.72 ± 19.70 ml		1235.28 ± 12.96 ml		1303.94 ± 15.50 ml		NA		NA	
		0.8 %		1.8 %	8.2 %	1.0 %	4.3 %	1.2 %	10.0 %				
9	Forearm, subject 2 1128.50 ± 9.76 ml	1128.50 ± 9.76 ml		1072.45 ± 33.66 ml		1054.53 ± 22.89 ml		1119.77 ± 23.29 ml		NA		NA	
		0.9 %		3.1 %	5.0 %	2.2 %	6.6 %	2.1 %	0.8 %				
10				1108.26 ± 16.11 ml		1096.81 ± 27.16 ml		1161.05 ± 26.89 ml		NA		NA	
				1.5 %	1.8 %	2.5 %	2.8 %	2.3 %	2.9 %				
11				1168.53 ± 28.68 ml		1136.85 ± 14.06 ml		1203.69 ± 12.81 ml		NA		NA	
				2.5 %	3.5 %	1.2 %	0.7 %	1.1 %	6.7 %				

*Measured value* result value including standard deviation, *RSD* relative standard deviation, *RACC* relative abs. accuracy relative to Reference value, *Reference value* volume computed from precise dimensions in case of reference cylinder, in case of other methods, the reference value is the volume measured using Water Displacement Method

^a^For detailed description of RACC and Reference Value for MRI and CT see “Accuracy and repeatability experiments” section

**Fig. 8:**
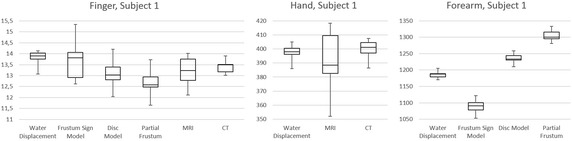
Results of comparison among tested methods performed on human subject 1

*In second step*, all the methods were tested on the patients. Two subjects, 26 and 39 years old, currently without pathological findings, were used for measurements of Forearm, Hand and Finger regions. Measurement of each region on every patient was also performed 10 times. Results of this step are shown in Table 1 in rows 2, 3, 5, 6, 8 and 9 and on Fig. 8.

Since aluminium objects are not allowed for CT and MRI, true values of measured volumes are not known, so reference values for assessment of CT and MRI accuracy were stated as follows: Water Displacement method and circumferential methods were examined in both first and second step and dependency between accuracy and measuring method was the same, so we can assume, that the most accurate method according to first step will be also the most accurate method in second step. Since this best accurate method, Water Displacement Method reaches up to 0.3 % accuracy, its value was taken as reference in case of analysing CT and MRI methods, where true value is not known.

*Accuracy* (relative absolute accuracy, RACC) was computed as relative difference between measured value of volume and true value of volume according to formula:

$$ RACC = \left| {\frac{{\bar{x} - x_{T}  }}{{x_{T} }}} \right| $$

where $\bar{x}$ is given as:

$$ \bar{x} = \frac{1}{N} \mathop \sum \limits_{i = 1}^{N} x_{i} $$

variable $x_{T}$ means a true value of volume, N is number of measurements (10) and $x_{i}$ is i-th measured value.

This parameter represents influence of systematic errors on measured value.

*Repeatability* (RSD) was computed as standard deviation of measured values in case of same object and same person performing the measurement according to formula:

$$ RSD = \frac{{\sqrt {\frac{1}{N}\mathop \sum \nolimits_{i = 1}^{N} (x_{i} - \bar{x})^{2} } }}{{\bar{x}}} $$

This parameter represents influence of random errors on measured value.

### Personal dependency test

In case of circumferential measurements, the result value of volume is dependent on particular person performing the measurement, since the value affects how much the string is tightened.

Three sets of 10 measurements on the same Forearm region using each circumferential method were performed by three different people in order to test personal dependency of the method. Results of this test are shown in Table 1 in rows 9, 10 and 11.

## Results

Overview of measured values is summarized in Table 1. Diagrams on Fig. 7 and on Fig. 8 show median, first and third quartile, minimal and maximal measured value for each method.

Water Displacement method can be considered as the best method overall, since it has the very best accuracy (RACC = 0.3 %) and the best repeatability (SD up to 0.9 % at Forearm) together with simplicity—no expensive equipment is needed, the apparatus directly shows the measured value and it is operator independent. Experimental accuracy (0.3 %) was better than accuracy estimated in (Kaulesar Sukul et al. 1993) (2 %). It was even better since the size of object was bigger.

It was observed, that the only problem of the method is shivering of the measured limb, what causes false dropping and consequently increase of measured volume. It is clear from SD, which is about 2 times lower in case of reference cylinders. This phenomenon has been observed more at the last measurements, when examined subject started to be tired. Measure procedure is also time consuming (15 min.) comparing to circumferential methods, because the dropping is very slow.

The Frustum Sign Model was very fast (less than 1 min.), but it was very personally dependent in both accuracy and repeatability. The repeatability was in range from 2 to 6 % and accuracy in range from 2 to 8 % according the operator. But difference in resulting value in case of measurements of the same object by various operators was up to 10 %. On the other side, the best single operator was able to measure with RACC = 1.8 % and SD = 1.5 %.

Both Disc Model and Partial Frustum Model were also personally dependent, but the repeatability was better (1–2 % Forearm, 3–4 % Finger). The best operators reach also better accuracy (up to 1 %). However, the measurement was significantly slower (15 min.). There is no proven difference between these methods, but Partial Frustum Model is preferred, due to its more authentic volume approximation, what could theoretically lead to better accuracy.

The MRI and CT based measurements were significantly more expensive, but there are the only methods, where selecting of special region is allowed. Since MRI has lower resolution and there is also more noise in MRI data, the edge of object is harder to detect precisely, so the repeatability was lower in case of MRI (5 %) compared to CT (2 %). Also the accuracy was very good in case of CT (2 % finger, 0.5 % hand). The CT is the only method, which reaches up to the same accuracy and repeatability as the Water Displacement Method; however its use has adverse effect on human health.

Overall summary of parameters of compared volumetric methods shows Table 2. It can be used as a guide when choosing the proper method for specific application.

**Table 2:** Overview of defining parameters of compared methods and their recommended usage

	Water Displ.	Frustum Sign	Disc Model	Partial Frustum	MRI	CT
Accuracy^a^	0.3 %	2–8 %^b^	1–8 %^b^	1–8 %^b,c^	Finger 8 % Hand 2 %	Finger 2 % Hand 0.5 %
Repeatability^d^	Finger 2 % Forearm 0.9 %	2–6 %^b^	Finger 3–4 % Forearm 1–2 %^b^	Finger 3–4 % Forearm 1–2 %^b^	5 %	2 %
Measure time	Finger 3 min Other 15 min	1 min.	Finger 8 min Forearm 14 min	Finger 8 min Forearm 14 min	Hand 19 min	Hand 7 min
Processing time	Less than 1 min	Less than 1 min^e^	1 min^e^	1 min^e^	12 min	7 min
Measurement cost^f^	Less than 1 EUR	Less than 1 EUR	Less than 1 EUR	Less than 1 EUR	250–300 EUR	100 EUR
Operator dependency^g^	None	High	High	High	Low	Low
Special region measurements^h^	No	No	No	No	Yes	Yes
Limitations	Shivering body, flexibility of limb, infection	Almost none			Claustrophobia, no metal (piercing, pacemaker,…)	High radiation dose, artefacts on metal parts
Recommended use	Best for accurate measurements of flexible and non-infected limbs. Good for standard use	When very fast, but not accurate (only estimation) of volume is required. Use the same staff to avoid operator dependency	No reason to use this method, same usage as Partial Frustum^c^	Use this, when better accuracy is required, but patient does not meet the limitations of Water Disp. Use the same staff	Use this in case of specific measurement region, where high accuracy is not necessary or in case of repeated measurements	Use this, when accurate measurement of specific region is required and the measurement is single shot only

^a^Accuracy is defined as relative deviation of mean value from reference value (see Accuracy and repeatability experimentssection)

^b^Strongly dependant on person performing measurements

^c^Approximation of volume is more authentic than Disc Model, so accuracy should be theoretically higher. However, no significant difference was experimentally detected

^d^Repeatability is SD of measurements performed by same staff (see “Accuracy and repeatability experiments ” section)

^e^In case of writing the values into the auto-computing computer worksheet

^f^Refers to all the expenses on the measurement, including acquisition cost proportion relevant to one measurement

^g^If resulting measured value depends on particular abilities of person performing measurement

^h^Allowing any irregular nonstandard region of interest

## Discussion

Megens et al. (2001) or Kaulesar Sukul et al. (1993) consider Water Displacement method as the best method based on its repeatability. This experiment is conform to this claim and besides reliability, it evaluates also accuracy related to the true value, what is also the best from tested methods (Fig. 7).

Sander et al. (2002), Taylor et al. (2006) or Meijer et al. (2004) declares high correlation among circumferential methods and Water Displacement method, but significant discrepancy between values of these method. Based on that, they stated it cannot be indicated which method is preferable. This experiment evaluated method’s values in relation with true value and proved, that Water Displacement values are the closest from true value.

We confirmed repeatability of Frustum Sign and Disc Model given by Deltombe et al. (2007) and evaluated accuracy, which is worse than Water Displacement’s. On the other hand, this method cannot be considered as useless, since it is much faster than Water Displacement. It depends, how accurate measurement is necessary, but in many cases, its accuracy can be sufficient.

There are modification of Frustum Sign Model called Disc Model and Partial Frustum model, which were originally introduced in order to improve the accuracy. We examined, that improvement of accuracy is insignificant, especially in contrast with increase of measure time. Because of that, there is no reason to use another circumferential method than Frustum Sign Model.

We experimentally proved and quantified the assumption of Armer and Ridner (2006) that measured value is dependent on skills of person performing measurement (Table 1, rows 9–11).

All these method provides volume of entire limb, not the volume of muscles or oedemas only, what is usually value, which is required. In cases of measurements of oedemas, we consider changes in muscle mass as negligible, likewise in case of measurements of muscle growth or atrophy, we neglect oedemas. But there are cases, where these neglects are inappropriate. From this reason, we introduced new methods for limb volumetry into the comparison, CT and MRI, which are, even though expensive, the only methods useful in such cases.

The strength of this study is comparison of wide range of methods, presently used in limb volume measurements. The benefit of this study is also evaluation of all parameters necessary to know, when deciding which method to use, including accuracy or measurement time.

The limitation of this study is, that true value of measured object is known only for reference cylinders and not for human subjects. Because of that, the accuracy of MRI and CT cannot be stated exactly (marked with asterisk in Table 1) and provided values depends on validity of condition given in section “Accuracy and repeatability experiments”.

Future studies should verify the validity of this condition on another reference object with known true volume, which is more relevant to human limb and measurable also with MRI and CT (phantom). It should also investigate the lover limb since oedemas preferably occur at the feet.

## Conclusion

The ideal volumetric method for upper limb should be accurate, repeatable, operator independent, simple, inexpensive and fast. Except the last one, the Water Displacement Method has all the abilities, so it is recommended as a standard method.

When use of Water Displacement is not possible (flexibility, shivering, infection, etc.), we recommend to use Partial Frustum Model, which takes the same time as Water Displacement, and reaches the satisfactory accuracy.

When accuracy is not the decisive factor and fast measurement is preferred, use Frustum Sign Method. It is the fastest method, which results (when taken by the same person) can be useful for fast estimation of volume.

In case of all circumferential method, be aware, that result value is very personally dependent, so only measurements taken by the same staff are comparable. Two measurements taken by different staff can vary by up to 10 %.

Due to expensive operation, the MRI and CT methods are recommended only in cases, when measurement of specific region is necessary (e.g. volume of particular muscle). When only single measurement (or very few) is necessary, use CT, which is less expensive and very accurate. In case of repeated measurements or when accuracy is not too important, use MRI, which has no harmful influence on patient’s health.
